# Scyllo-Inositol as a Neuroactive Agent: From Pharmacokinetics to Neuroprotective and Antiepileptic Effects

**DOI:** 10.3390/nu18121955

**Published:** 2026-06-17

**Authors:** Karol Wiśniewski, Kamila Zglejc-Waszak, Aleksander Warzecha, Marcin Jozwik, Michael Thoene, Joanna Wojtkiewicz

**Affiliations:** 1 Students’ Scientific Club of Pathophysiologists, Department of Human Physiology and Pathophysiology, School of Medicine, University of Warmia and Mazury, 10-082 Olsztyn, Poland; wisniewski.karol@op.pl (K.W.); alesander.warzecha@student.uwm.edu.pl (A.W.); 2 Department of Human Physiology and Pathophysiology, School of Medicine, Collegium Medicum, University of Warmia and Mazury in Olsztyn, 10-085 Olsztyn, Poland; 3 Department of Anatomy and Histology, School of Medicine, Collegium Medicum, University of Warmia and Mazury in Olsztyn, 10-085 Olsztyn, Poland; kamila.zglejc@uwm.edu.pl; 4 Department of Gynecology and Obstetrics, School of Medicine, Collegium Medicum, University of Warmia and Mazury in Olsztyn, 10-045 Olsztyn, Poland; marcin.jozwik@uwm.edu.pl; 5 Department of Medical Biology, School of Public Health, University of Warmia and Mazury in Olsztyn, 10-561 Olsztyn, Poland; michael.thone@uwm.edu.pl

**Keywords:** scyllo-inositol, cyclitols, neuroprotection, pharmacokinetics, epilepsy, zebrafish, rat model

## Abstract

Neurodegenerative disorders and epilepsy remain major clinical challenges, due to complex etiologies involving protein misfolding, excitotoxicity, metabolic dysregulation, and impaired cellular resilience. These unmet medical needs have stimulated interest in small-molecule modulators capable of targeting multiple pathogenic pathways. Cyclitols, a diverse family of inositol stereoisomers, play essential roles in cellular signaling and brain metabolism; among them, scyllo-inositol (SCI) has gained attention due to its distinct stereochemistry, capacity to cross the blood–brain barrier, and emerging neuroactive properties. Current pharmacokinetic data indicate that SCI exhibits dose-dependent systemic exposure, and good penetration into the central nervous system. Moreover, its supplementation seems to be well-tolerated. In experimental studies both on animals and humans, SCI has been shown to modulate amyloid-β aggregation, stabilize neuronal homeostatic pathways, and reduce network hyperexcitability, suggesting relevance for both neurodegenerative and epileptic phenotypes. Despite promising results, there is still a need for further analyses to define dosing, transporter involvement, and brain exposure thresholds. Collectively, the available data position SCI as a compelling candidate for translational development, warranting further investigation into its therapeutic window and disease-modifying potential across neurological disorders.

## 1. Introduction

Cyclitols, are a class of naturally occurring compounds, which are based on a cycloalkane chain that contains at least three hydroxyl groups, each attached to a different ring carbon.

The most dominant cyclitols in eukaryotic cells are inositols (INSs) [1,2]. Cyclitols serve a key role in cellular physiology, especially in the central nervous system (CNS). INS stereoisomers contribute to signal transduction, osmoregulation, membrane biogenesis, ion channel physiology and have antioxidant activity in the brain [1]. INSs also regulate neuronal and glial activity, underlining the critical role of these molecules in maintaining neural homeostasis [3].

Scyllo-inositol (SCI) is quite widespread throughout biological systems. One of the largest concentrations of SCI (about 0.5 g/L) can be found in coconut milk, which has been successfully used as a growth-promoting agent in formulations of plant cell culture medium [4]. SCI can also be found in high amounts in legumes and cereals [5]. Furthermore, SCI has been identified in mammalian tissues, especially in the brain, kidney, and lens tissue [6]. Microorganisms such as Bacillus subtilis and Rhizobium species have been shown to both produce and utilize SCI [7,8,9]. Certain bacteria, including Streptomyces griseus, are capable of converting myo-inositol (MI) to SCI [10].

SCI has a symmetrical structure in which all six hydroxyl groups are oriented equatorially. This confers chemical stability and unique physicochemical properties such as high aqueous solubility and low reactivity towards nucleophilic substitution [11,12]. In chemistry, SCI is usually prepared from MI through a multi-step process by a Mitsunobu reaction [12]. SCI can also be synthesized from para-benzoquinone via a conduritol intermediate [13]. There are reports suggesting that engineered microbial systems expressing inositol dehydrogenases can efficiently biosynthesize SCI from inexpensive substrates [14,15].

Over the past three decades SCI has attracted growing interest as a neuroactive cyclitol with potential therapeutic relevance in a range of both neurological and neurodegenerative disorders. SCI has been widely tested as a therapeutic option in Alzheimer disease (AD) [16], Huntington’s disease [17] and Parkinson disease [18]. In each of these disorders, SCI supplementation showed benefits in both in vitro and in vivo animal trials. Moreover, SCI has been shown to decrease seizure in rat and Zebrafish models of epilepsy [19,20,21]. However, it is worth mentioning that despite promising preclinical results in the afore-mentioned neurological disorders, only one AD-randomized clinical trial has been reported [22]. It is also worth mentioning that although many pharmacokinetic studies were performed, the pharmacokinetics of SCI still have not been fully monitored, especially during longer supplementation. Moreover, important information about the molecular processes involving SCI and the way SCI plays its biological activity is still missing. Taking all of the above facts into consideration, there is a strong and growing need for translational studies to allow for a full understanding of the potential use of SCI.

The aim of this work is to integrate both the available pharmacokinetic and anticonvulsant data on SCI into a review.

## 2. Materials and Methods

This narrative review was conducted using a structured literature search strategy to identify relevant studies. Literature searches were performed in the PubMed and Scopus databases from database inception until December 2025.

The search strategy employed combinations of the following terms: “scyllo-inositol”, “inositol”, “cyclitols”, “pharmacokinetics”, “neuroprotection”, “epilepsy”, “seizure”, “antiepileptic”, “Alzheimer disease”, “Huntington disease”, “Parkinson disease”, “zebrafish”, and “rat model”. Operators (AND, OR) were used to combine search terms and optimize the retrieval of relevant publications.

The literature search, title and abstract screening, and full-text assessment were independently conducted by two authors. Any discrepancies regarding study eligibility or relevance were resolved through discussion and by consultation with other co-authors.

Inclusion criteria were research articles, clinical studies, experimental animal studies, and review papers investigating SCI or related ins stereoisomers in the context of pharmacokinetics, tissue distribution, transport mechanisms, neuroprotection, neurodegenerative disorders, epilepsy, seizure models, and associated molecular pathways. Additional references providing background information on epilepsy pathophysiology, neurodegenerative diseases, and experimental animal models were identified through manual screening of reference lists of selected articles.

Studies were excluded if they were unrelated to the neurological, pharmacological, or biological effects of SCI, were duplicates, lacked sufficient methodological information, or were not available in English.

The chosen studies were selected based on their scientific relevance, methodological quality, and contribution to understanding the pharmacological and neurobiological effects of SCI. The final review included 127 references comprising available knowledge about SCI and its properties.

## 3. Chemical Structure and Biological Properties of Scyllo-Inositol

**Figure 1:**
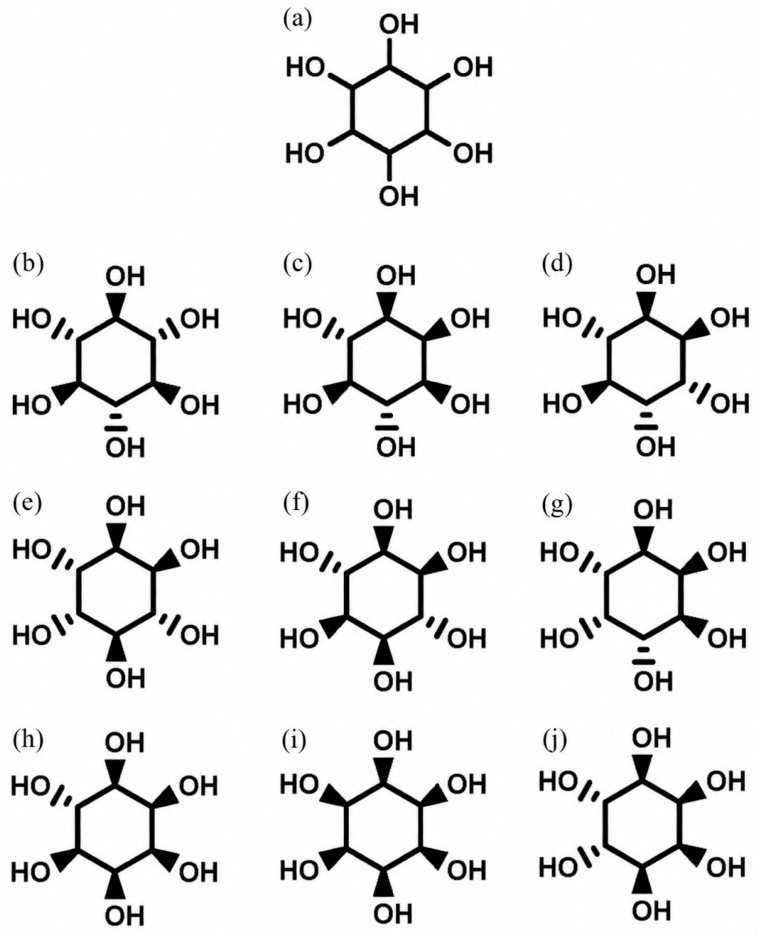
Structural formulas of (**a**) inositol, (**b**) scyllo-inositol, (**c**) myo-inositol, (**d**) D-chiro-inositol, (**e**) L-chiro-inositol, (**f**) muco-inositol, (**g**) neo-inositol, (**h**) epi-inositol, (**i**) cis-inositol, (**j**) allo-inositol.

SCI, also known as scyllitol or cyclohexane-1,2,3,4,5,6-hexaol, is one stereoisomer of INS (Figure 1). The molecular formula of SCI is C_6_H_12_O_6_ and the molecular mass is 180.156 g/mol. SCI has a ring of six carbon atoms, each bound to one hydrogen atom and one hydroxyl group. Its molecular conformation adopts a chair-form in which all six hydroxyl (–OH) substituents are placed equatorially [23]. Although most literature suggests the existence of nine inositol isomers (SCI, MI, neo-inositol, epi-inositol, muco-inositol, cis-inositol, allo-inositol, L-chiro-inositol, and D-chiro-inositol(DCI)) [6], theoretical studies suggest that even thirteen INS isomers are possible [23]. Anhydrous SCI crystallizes in at least two polymorphic forms: the “A” form (monoclinic, with a density ca. 1.57 g/cm^3^) and the “B” form (triclinic, with a density ca. 1.66 g/cm^3^). The water solubility of SCI is estimated to be ~10 g/L (~10 mg/mL) at ~18 °C. The melting point of solid SCI has been reported as roughly 350 °C [24].

SCI, with its equatorial orientation of all hydroxyl groups, has the shortest carbon–carbon(C-C) average bond length in comparison to other stereoisomers, which promotes a chair-like structure. Moreover, the average CCC angle in SCI is also smaller than in other INSs, which indicates a small degree of planarity in all isomers except SCI [23].

The presence of intramolecular hydrogen bonding in INS is supported by various studies [18,25]. The results indicate that the intramolecular hydrogen bond involving axial hydroxyl groups is stronger than equatorial ones. Thus, the axial hydroxyl group actually provides a stabilizing effect, which may be the reason for lower internal energies obtained for certain inositol isomers as compared to SCI [23].

SCI is considered to be the most stable inositol in polar solvents, because of favorable intermolecular solute–solvent interactions relative to other axial–OH groups containing INSs [23]. The hydroxyls in SCI are better positioned to form intermolecular bonds [26]. Weaker intramolecular OH-O hydrogen bonds in SCI between equatorial hydroxyl groups also increase stability due to more favorable intermolecular interaction. However, in the gas phase, other INS stereoisomers with axial hydroxyl groups (like myo-ins, neo-ins) are more stable than INS [23].

The analysis of zero-point vibrational energy (ZPE) reveals that SCI is the most stable. In the case of entropy or thermal energies, other isomers are favored over SCI. However if overall energies are considered, by inclusion of electronic energies along with thermal energies, then only myo- and neo-inositol seem to be more relatively stable than SCI. In the case of free energies of INS, SCI shows stability over all other isomers [23].

INS derivatives play important roles in various biological activities. Moreover, amino or substituted amino derivatives of INS also occur in many antibiotics [27,28]. “Insoamino acids’’ are a new class of inositol derivatives composed of INS and b- or g-amino acids, used for the construction of peptide nanotubes with hydrophobic cavities [29]. However, it is worth mentioning that regioselective chemical modifications (e.g., selective esterification or etherification) of SCI are challenging. The reason for that is because each hydroxyl site is equivalent, without special protecting-group strategies one cannot easily distinguish or selectively derivatize particular positions. Furthermore, synthesization of SCI derivates require global substitution or careful protection [30]. It is also worth mentioning that due to its internal symmetry SCI is optically inactive. The SCI spectroscopic signature is much simpler when compared to other INS isomers, which allows it to be used in proton nuclear magnetic resonance (NMR) for SCI detection and quantification in vivo [30].

## 4. Pharmacokinetics of Scyllo-Inositol

The easiest and most well-tolerated method of INS supplementation appears to be oral [31]. Although the mechanisms of SCI uptake and metabolism are not fully understood, knowledge about the pharmacokinetics of MI, which has many similarities to SCI, is well established. It is known that after oral administration, MI is almost fully (in 99.8%) absorbed in the human gastrointestinal tract due to active transport in a Na^+^-dependent manner [32]. There is also evidence that sugars (for example, glucose) can significantly reduce MI uptake in a noncompetitive manner [6]. MI can be converted to SCI by epimerases [33] and both SCI and MI can be transported by the same transporters [34,35]. It is also known that some tissues, for example brain, have about 100-fold greater concentrations of SCI and MI than found in blood [30,36]. MI may be produced endogenously in the human kidney and liver from glucose-6-phosphate [37]. The kidney is also the main organ where the catabolism of MI takes place [38].

The transport of SCI into the CNS appears to depend on active transport across the blood–brain barrier rather than simple passive diffusion. It is known that two sodium-dependent inositol transporters have been identified in brain: SMIT1 and SMIT2. Both of them were shown to transport SCI and MI in an active way [39]. The oral supplementation of SCI was shown to increase SCI brain concentrations; thus administered SCI penetrates and accumulates in CNS [40]. However, elevated SCI levels after supplementation did not result in incorporation of SCI into phosphatidylinositol lipids, suggesting that once transported into the CNS, SCI remains in its free form rather than being metabolized into membrane-associated inositol lipids, which distinguishes SCI from many other INS stereoisomers [41].

In our previous study, we tested the SCI blood concentration in rats after oral administration of 10 mg/kg of SCI [42]. This trial revealed that the first measurement of SCI concentration was recorded 30 min after administration. The maximum concentration (Cmax) of SCI was reached at one-and-a-half hours after administration. A second peak of SCI was not observed. SCI concentrations were undetectable 12 h after oral administration, although the MRT was determined to be 14.52 h. The total drug exposure integrated over time was 23.47 mg·h/L. There were no side effects of SCI supplementation noticed during the study [42].

In 2010, Choi JK et al. tested mice SCI brain concentrations after oral supplementation in mouse models of AD. The SCI was supplemented in water in the amount of 3.3 mg/kg/day for 2 months. The results of this trial showed a 3.0 fold increase and a 2–2.4-fold increase in SCI in brain extracts respectively in SCI-fed mice compared to normal diet. This was also seen in high resolution magic angle spinning. Moreover, SCI treatment was associated with an increase in glutamine in the hippocampus. The SCI concentrations were noticed to be higher in the hippocampus than in the frontal cortex [40].

In 2013, Yamashita et al. [43] made a comparison between the plasma concentration of SCI, MI and DCI, one hour after oral supplementation. In this trial, mice were orally administered 1000 mg/kg of inositol isomers. The results of the experiment showed that SCI concentrations were significantly higher than others, which may suggest that absorption of MI and DCI is either less efficient or that their excretion/cell incorporation is higher. After SCI supplementation, MI plasma concentration was also higher, suggesting that SCI is converted in vivo to MI. However, with the change in SCI dosage (500 mg/kg, 1000 mg/kg, 2000 mg/kg) no significant change in MI plasma concentration between the groups was noticed, opposite to the proportional change in the SCI plasma level. This might suggest that the conversion of SCI to MI is limited or controlled by unknown mechanisms [43].

The pharmacokinetics of SCI in humans was tested by Liang and co-workers in 2013 [44]. In this trial, eight healthy adult men received oral doses of 2000 mg of SCI twice a day for 10 days. The results showed that after administration of the first dose, SCI plasma concentration rapidly increased, reaching its maximum (17.2 mg/mL) at 3.8 h post-dose. Afterwards, the peak SCI concentration began to drop. The steady state of SCI concentration was reached by day 5. After the last dose of SCI, a maximum concentration of 39.8 mg/mL was reached 2.9 h post-dose, and then began to decrease rapidly. The AUC during each dose interval increased from 98.9 h mg/mL on day 1 to 277.0 h mg/mL on day 11, respectively. Moreover, Cmax and AUC values of SCI in CSF increased from 6.5 mg/mL and 49.4 mg·h/L on day 1 to 13.7 mg/mL and 153.0 mg·h/L on day 11, respectively. The brain concentration of SCI also increased (by 58–76%) during the study. All of these results implicated the accumulation of SCI during the study. It is worth noticing that no side effects of SCI supplementation were noticed during this trial [44].

In another clinical trial, conducted on patients with mild to moderate AD, doses of 250 mg, 1000 mg and 2000 mg of SCI were supplemented orally twice a day. The study level of plasma SCI was reached between week 2 and 12. CSF concentrations measured at week 24 were 13.8 μg/mL in the 250 mg group, 31.4 μg/mL in the 1000 mg group, and 35.1 μg/mL in the 2000 mg group. However, it should be pointed out that due to an imbalance of infections in the 2000 mg group and deaths in both 1000 mg and 2000 mg groups at 48 weeks, the two higher-dose groups were electively discontinued [22].

## 5. Neuroprotective Potential of Scyllo-Inositol

Neurodegenerative disorders are characterized by progressive loss of selectively vulnerable populations of neurons [45,46]. Neurodegenerative disorders can be classified in different ways; however the most common one involves a molecular abnormality. The most widespread neurodegenerative disorders are amyloidoses (e.g., Alzheimer’s disease (AD), tauopathies (e.g., Pick’s disease), α-synucleinopathies (e.g., Lewy body disorders), and TDP-43 proteinopathies (e.g., Primary lateral sclerosis) [45]. Diagnosis of neurodegenerative disorders are involved in the clinical prevalence of the patient and supportive evidence for additional diagnostics (e.g., magnetic resonance imaging or electroencephalography) [47,48,49]. Although there are many approved therapies to address neurodegenerative disorders, most of them only help to treat the symptoms and do not influence the progression of the disorder [50].

### 5.1. Alzheimer’s Disease

AD is a progressive neurological disorder that primarily affects the brain, leading to a decline of memory, cognitive abilities, and behavioral patterns. AD is the leading cause of dementia, which is estimated to affect over 50 million people worldwide [51], and could even triple by 2050 [52]. AD pathophysiology includes the accumulation of abnormal protein aggregates in brain—amyloid-β plaques and intracellular tau neurofibrillary tangles [53]. These deposits lead to a derangement of impulse passage, cell death and atrophy of brain tissue [54].

Currently, the treatment of AD involves both nonpharmacologic approaches (such as psychotherapy, behavioral therapy or physiotherapy) [50,55] as well as pharmacological therapies [56,57,58]. Although such approaches can decrease symptoms and slow down cognitive decline, it cannot stop the development of AD [50].

The SCI journey as a possible therapeutic option started in 1996, when McLaurin & Chakrabartty [59] showed that gangliosides in neutral pH induce Aβ40/42 to adopt a novel α/β conformation, which may sequester Aβ and thereby prevent β-structured fibril formation [59]. Further in vitro studies supported these findings [16].

Encouraging results were subsequently reported in animal models. Townsend et al. demonstrated that oral SCI administration attenuated memory impairment induced by intracerebroventricular injection of soluble Aβ oligomers in rats, suggesting that SCI may restore hippocampal synaptic plasticity disrupted by Aβ toxicity [60]. Similarly, McLaurin et al. showed that prophylactic SCI administration in TgCRND8 mice reduced plaque burden, neuroinflammation, and synaptic toxicity while improving survival [61]. Additional studies in PS1xAPP and 5XFAD mice confirmed reductions in amyloid plaques and improvements in synaptic integrity as well as cognitive performance [62,63]. However, treatment with a combination of SCI and *R*-flurbiprofen was not more effective than with SCI alone. Moreover, SCI-treated mice performed significantly better at the radial arm water maze task than either the placebo or SCI + *R*-flurbiprofen group [63]. Collectively, these studies consistently demonstrated beneficial effects of SCI across several transgenic AD models.

The very promising results from in vitro and in vivo studies have led to clinical trials. In 2011 a randomized, double-blind, placebo-controlled, dose-ranging phase 2 study was carried out. The aim of the study was to explore the safety, efficacy, and biomarker effects of SCI oral supplementation in mild to moderate AD. The study was conducted on 353 patients, who were randomized to 250, 1000, or 2000 mg SCI groups or placebo. The study duration was 78 weeks. Due to an imbalance of infections in the 2000 mg group and deaths in both the 1000 mg and 2000 mg groups, the two higher-dose groups were electively discontinued. Nine of ten deaths in the study (0, 1, 5, and 4 deaths in the placebo, 250, 1000, and 2000 mg groups, respectively) were assessed as not related to the study drug by the reporting investigator. The 250 mg SCI dose demonstrated acceptable safety, however no significant benefits of SCI supplementation were noticed [22]. The summary of SCI effects in AD is presented in Table 1.

The apparent discrepancy between preclinical findings and subsequent clinical outcomes highlights a substantial translational gap. Animal models of AD reproduce selected pathological features, particularly amyloid accumulation, but incompletely capture the multifactorial nature of human disease, including tau pathology, vascular dysfunction, neuroinflammation, and age-related comorbidities. Furthermore, reductions in amyloid burden do not necessarily translate into clinically meaningful cognitive benefits. It is also worth noticing that, although investigators concluded that most of these adverse events were not directly attributable to SCI, their occurrence raises important questions regarding the safety of sustained high-dose exposure in a vulnerable elderly population. Notably, pharmacokinetic analyses indicated non-linear SCI disposition, with cerebrospinal fluid exposure increasing disproportionately relative to plasma concentrations. This observation suggests that CNS exposure may not have easy predictably and could contribute to a narrow therapeutic window. Moreover, the lack of significant positive outcomes, despite increased SCI CNF concentration, may suggest that amyloid modulation was not significantly increased or, alone, may be insufficient to change the natural course of AD.

Future studies should focus on identifying optimal exposure ranges, clarifying the clinical implications of SCI’s non-linear pharmacokinetics, and determining whether earlier intervention stages or combination approaches may offer greater therapeutic potential.

### 5.2. Other Neurodegenerative Disorders

Building on growing evidence implicating the use of small molecules to inhibit aggregation of amyloidogenic proteins [66,67], Ibrahim and McLaurin in 2016, investigated in vitro properties of SCI of inhibition of alpha-synuclein aggregation [18]. This small protein is enriched in synapses where it participates in synaptic vesicle functioning [68]. However, due to both environmental factors and genetics, alpha-synuclein can aggregate and bypass normal clearance which can cause synucleinopathy in the brain, which is one of the underlying pathogenic causes of Parkinson’s disease (PD) [69,70,71,72]. In this trail [18], the authors incubated both human (0.5 mg/mL) and mouse alpha-synuclein (0.1 mg/mL) as monomers with both SCI and chiro-inositol. The results of this experiment showed that SCI prevented fiber formation in both human and mouse alpha-synuclein. However, co-incubation of alpha-synuclein and chiro-inositol did not provide any benefits [18].

Motivated by the need for disease-modifying strategies in Huntington’s disease, Lai and colleges (2013) [17] conducted a study in which Htt14A2.5 PC12 cells (a cellular model of Huntington disease) were treated with SCI. The results showed a significant dose-dependent reduction in visible aggregates in SCI concentrations ranging from 10 up to 100 µM. At 100 µM of SCI, the decrease in cell-containing aggregates was 15%, which is comparable to known inhibitors of huntingtin protein aggregation—cystamine (in concentration of 50 µM) [73]. Furthermore, the authors in a series of experiments showed that SCI effects were mediated by the induction of chaperone-mediated autophagy degradation of polyQ-Htt. Worth mentioning is the fact that no signs of SCI toxicity were detected during the study. Altogether, these results suggest that SCI promotes mutant huntingtin degradation in a model of Huntington disease [17].

## 6. Antiepileptic Properties of SCI

Epilepsy, defined as a state of recurrent, spontaneous seizure [74], is one of the biggest challenges in medicine nowadays. It is estimated to affect over 70 million people worldwide [75]. It is believed to result from pathological hyperexcitability within neuronal populations due to a disruption in the balance between inhibition and excitation [76,77]. The underlying cause of epilepsy is often not clear, but the most common ones are: brain injury, toxic event, infection, stroke, brain tumor, developmental abnormalities and genetic disorders [78,79].

The pathogenesis of epilepsy involves some complex mechanisms involving ion balance, both in the cellular and extracellular space lm [80,81,82,83], glia function [80,84], synaptic transmission [85] and synchronization of neuronal activity [86,87].

Although there are many commercially available antiepileptic drugs, about 30% of all patients do not respond to any of them [88]. For such patients, surgery or neurostimulation may be a therapeutic option [85,89]. However, even these methods are not effective in all patients with observed recurrent seizures [85].

Experimental models, particularly zebrafish and rodent models, have become important tools for studying epilepsy and screening potential therapeutic agents. Zebrafish are especially useful due to their similarity in brain organization with other vertebrates [90], mapped neurotransmitter systems [91], genetic similarity to other vertebrates, and measurable neurobehavioral traits [92,93]. In epilepsy research, seizures are commonly induced using pentylenetetrazol (PTZ), a GABA-A receptor antagonist that produces dose-dependent neuronal hyperexcitation through inhibition of GABAergic neurotransmission, leading to dose-dependent seizures [94,95,96,97,98].

Currently available evidence suggests that INSs may possess antiepileptic and antiepileptogenic properties. Initial studies in zebrafish larvae demonstrated that short-term SCI exposure reduced PTZ-induced hypermobility in all tested groups, compared to controls. However, in the long-term exposure protocol, no significant changes were observed, which may be due to the use of insufficient SCI dosages to produce therapeutic effects [19]. Similar findings were obtained in rodent models, where both SCI and MI reduced seizure severity, seizure duration, and increased seizure latency in PTZ-induced seizures [20]. Furthermore, evidence from kainic acid (KA)-induced epilepsy rodent models demonstrated that MI, SCI, and D-chiro-inositol (DCHI) reduced the frequency and duration of spontaneous recurrent seizures and improved cognitive deficits associated with epileptogenesis [21]. Importantly, these effects persisted for several weeks after treatment discontinuation, suggesting potential disease-modifying properties rather than only symptomatic seizure suppression [21]. Moreover, there is also evidence that SCI, MI and DCHI supplementation induces change in the concentration of several proteins [21]. These proteins, for example, alpha-synuclein, deglycase DJ-1 (parkin-7), 14-3-3proteins, or dihydropyrimidinase-related proteins, are implicated in neurotransmitter release at neuronal synapses [68], oxidative stress and glycation [99], neuroprotection [100], signal transductions and cellular diversion processes [101].

The antiepileptic potential of MI, the precursor of SCI, has been demonstrated across different experimental paradigms. In rodent models, intracerebroventricular administration of MI prolonged seizure latency and reduced seizure severity in Li+-pilocarpine-induced seizures [102]. Furthermore, systemic administration reduced seizure activity in both PTZ- and KA-induced models [103]. Additional studies confirmed that hippocampal administration of MI significantly shortened epileptiform discharge duration [104]. There is also evidence that long-term MI treatment reduces seizure frequency and duration, attenuate neuronal cell loss, improve spatial learning and memory, and induce molecular changes in the hippocampus, including altered miRNA and protein expression profiles in rodent models [105,106]. The summary of INS action in epilepsy is presented in Table 2.

Taken together, the available experimental evidence supports the hypothesis that both SCI and MI may act not only as symptomatic anticonvulsant agents but also as potential disease-modifying compounds capable of influencing the progression and neurological consequences of epilepsy.

Currently, the SCI mechanism of action is unclear and there are only a few molecular explanations for MI’s antiepileptic effects. It has been reported that MI and SCI demonstrate many similarities in terms of both structure and mode of action [6]. MI is the precursor of SCI and a specific epimerase converts MI to SCI [107,108]. MI as well as SCI are both taken up into cells by the same active transporters [109].

The MI antiepileptic properties seem to be mediated through a couple of mechanisms. The first one is the modulation of GABA and NMDA receptors. There is evidence that in vitro MI stimulates 3H MK-801, a specific ligand of activated NMDA-type glutamate receptors and inhibits 3H muscimol, a specific ligand of the GABA-A receptor [110]. Furthermore, the α4 and γ2 subunits of GABA-A receptors seem to be restored to their normal level after MI treatment in comparison to ones treated only with KA [111]. It is worth pointing out that the α4 subunits are crucial for tonic inhibition and are predominantly expressed in extrasynaptic location [112], whereas γ2 is found in synapses and participate in phasic inhibition [113]. These findings are further supported by recent proteomic analyses demonstrating that inositol supplementation affects proteins involved in synaptic signaling and neurotransmitter regulation [21]. Moreover, given the established role of phosphoinositide signaling in receptor trafficking and membrane dynamics [114], it is conceivable that MI may indirectly influence GABA-A receptor localization and downstream signaling pathways, although direct experimental evidence supporting this mechanism is currently lacking. In addition, increasing evidence suggests that astrocytes play a critical role in maintaining extracellular GABA concentrations and regulating inhibitory neurotransmission [115,116]. Furthermore, NMDA receptor activity plays an important role not only in seizure initiation but also in synaptic plasticity and long-term epileptogenic remodeling, suggesting that regulation of glutamatergic neurotransmission may have both acute anticonvulsant and long-term antiepileptogenic consequences [117,118]. Taking all of this into consideration, the available evidence suggests that modulation of GABAergic and glutamatergic neurotransmission, in particular by modulatory action on the respective postsynaptic receptors, may contribute to the anticonvulsant effects of inositols [111]

The other possible mechanism involves MI osmotic activity. During an increased rate of neuronal activity, a massive influx of Na^+^, Ca^2+^, Cl, and water occurs which may produce cellular swelling resulting in disturbances of enzyme activity and functions. MI, as an osmolyte, can contrapose these processes leading to proper neuronal function [119,120,121]. The third possible mechanism is a modulation of ion channels. Increased intracellular MI concentration has been shown to increase polyphosphoinositide levels and to modulate neuronal activities via PI(4,5)P2-dependent ion channels [122].

In 2025, Tsverava et al. [21] suggested an interesting theory, that due to much smaller amounts of SCI and DCHI needed to observe therapeutic effects (in comparison to MI 6 and 30 times respectively) and the fact that MI is a converter in the organism to SCI and DCHI, it is possible that some of the MI antiepileptic properties are mediated by increased levels of SCI and DCHI. Further studies are needed to fully understand the mechanisms of SCI and MI action.

**Figure 2:**
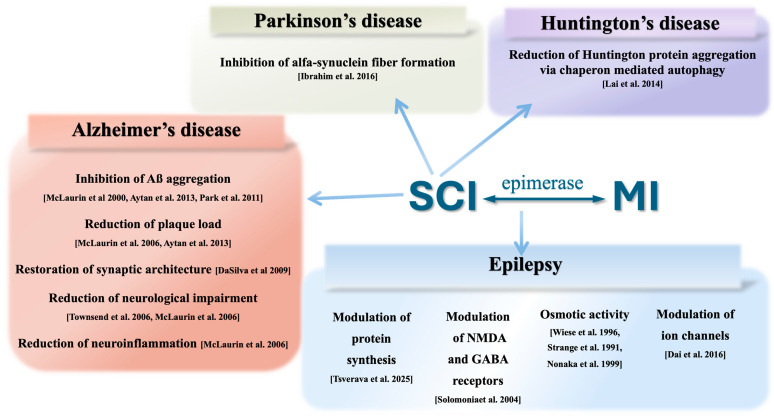
Summary of SCI neuroprotective and antiepileptic properties [16,17,18,21,60,61,62,63,64,110,119,120,121,122]. Abbreviations: scyllo-inositol (SCI), myo-inositol (MI), N-methyl-D-aspartate (NMDA), gamma-aminobutyric acid (GABA), Amyloid-beta (Aβ).

Overall, current evidence supports a protective and potentially disease-modifying role of inositols in epilepsy. Experimental studies consistently demonstrate anticonvulsant, neuroprotective, and cognition-preserving effects of SCI and MI across different seizure models and species [2,19,20,21,102,103,104,105,106] (Figure 2). These findings are particularly encouraging when taken into consideration the fact of limited availability of therapies capable of modifying epileptogenesis rather than only suppressing seizures symptomatically. Moreover, the multimodal mechanisms proposed for INS suggest that these compounds may target several pathological processes simultaneously. Nevertheless, despite these promising preclinical observations, the translational potential of SCI and MI remains uncertain. Importantly, many studies were conducted on relatively small experimental groups and focused primarily on short-term outcomes, while long-term safety and efficacy data remain unknown. Moreover, it must also be taken into consideration that epilepsy is a heterogeneous group of disorders with distinct etiologies, molecular mechanisms, and clinical manifestations, which would probably affect INS efficiency. Another important limitation concerns the pharmacokinetic and pharmacodynamic properties of INS in humans and the safety of doses needed to obtain a significant antiepileptic effect. Finally, it should be pointed out that the above-mentioned studies compered INS effects to placebo, not standardized antiepileptic drugs, which should also be taken into consideration.

**Figure 3:**
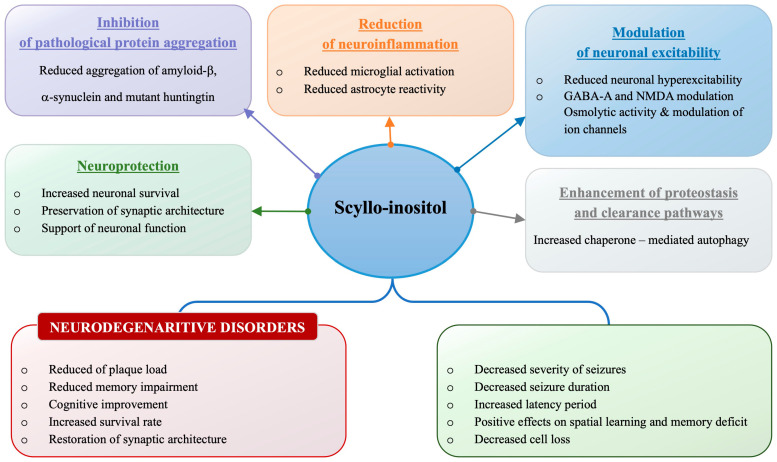
Summary of SCI properties. Abbreviations: N-methyl-D-aspartate (NMDA), gamma-aminobutyric acid (GABA).

Taken together, SCI and MI should currently be regarded as promising experimental candidates rather than clinically validated antiepileptic agents (Figure 3). Well-designed translational studies and randomized clinical trials are necessary to determine their efficacy, optimal dosing strategies, safety profile, and potential role as adjunctive therapies in human epilepsy treatment.

## 7. Translational Relevance and Clinical Perspectives

The translational development of each new substance with possible therapeutic use critically depends on its pharmacokinetic characterization. It is a prerequisite for optimal dose selection to achieve the optimal therapeutic effects. The safety and side effects of substance administration need to be determined before further clinical application. Pharmacokinetic studies aim to provide information about different routes of substance administration, which is crucial for further trials in humans.

SCI pharamakokinetics has been a field of interest for some studies. It is currently known from clinical practice that the dosage of 250 mg of SCI twice a day, administered oraly for a prolonged period of time (78 weeks) turned out to be safe and well-tolerated. It is also known that a dosage of 1000 mg or more twice a day does not offer enough safety for a long supplementation period [22]. However, in short term studies (10 days) a dosage of even 2000 mg of SCI twice a day provided very good tolerance without side effects [44], which shows the importance of long-term studies. It is also worth pointing out that the short term study mentioned above was performed in a group of young male patients in comparison to patients aged 50–85 in a long-term study.

It is also worth mentioning that the SCI CSF concentrations measured in long-term studies at week 24 were 13.8 μg/mL in the 250 mg group, 31.4 μg/mL in the 1000 mg group, and 35.1 μg/mL in the 2000 mg group [22]. Notably, the increase in CSF exposure between 1000 mg and 2000 mg was modest compared to the substantial rise observed between 250 mg and 1000 mg, suggesting a non-linear relationship between dose and CNS penetration at higher dose ranges. The dosage increase from 250 to 1000 mg (four times) resulted in an over 2,2-fold increase in SCI CSF concentration, whereas the increase in dosage between 1000 and 2000 mg increased the SCI CSF concentration only for about 10%. This apparent plateau in CSF concentration at 2000 mg may reflect partial saturation of transport mechanisms across the blood–brain barrier, limited solute permeability, or compensatory clearance processes within the CNS. Taking into considaration these data, the SCI dosage between 250 and 1000 mg twice a day appears particularly relevant, because increasing dosage in this range will result in the biggest increase in SCI CSF concentration without unnecessary systemic exposure associated with higher dosing. Furthermore, given the potential for dose-dependent adverse effects and the need for long-term administration in chronic neurological disorders, identifying the lowest effective dose that achieves therapeutically relevant CNS concentrations is of critical clinical importance. Collectively, these data support the systematic investigation of 250–<1000 mg of SCI administered twice daily as a rational dosing window for further pharmacokinetic characterization and efficacy evaluation in clinical studies in different age groups.

SCI has shown positive effects in many disorders. The main point of interest for its possible clinical application seems to be neurodegenerative disorders and epilepsy. Together, more than 120 million people worldwide are suffering from these diseases. In this group, there are many whose treatment is insufficient. For these patients, particularly in drug resistant epilepsy, SCI may be considered a therapeutic option. In animal models of epilepsy, SCI supplementation decreased seizure duration, intensity and frequency [19,20]. Moreover, SCI mitigates the spatial learning and memory deficits associated with epileptogenesis [21], and the latency period was increased after SCI administration [20]. These results all support SCI as a possible antiepileptic agent. However, so far no clinical studies of SCI supplementation in human epilepsy, have been performed, which is a gap to fill for further research. When it comes to neurodegenerative disorders, the only one in which a clinical trial was reported was AD. In this trial, although a reduction in CSF Aβx-42 level was noticed, no significant changes in patient functioning or cognitive function were noticed. The only dosage used for the analysis was the 250 mg group (due to the discontinuation of the 1000 and 2000 mg groups) [22]. Taking into consideration the available above-mentioned data about SCI CSF concentration, there is still a need for further evaluation of dosages between 250 and 1000 mg. Further studies are needed to better define the dosage of SCI in AD. There are also some positive preclinical data in other neurodegenerative disorders [123].

Nevertheless, the translation of promising preclinical findings into successful clinical interventions remains one of the major challenges in scientific research. Several factors may contribute to the discrepancy between preclinical and clinical outcomes. First, it must be pointed out that although animal models are very helpful to understand and study disorders, unfortunately they can only partially reproduce the complexity of human diseases. In epilepsy research, for example, experimental models are typically based on acute seizure induction, whereas human epilepsy comprises a highly heterogeneous group of disorders with diverse etiologies, disease courses, genetic backgrounds, and response to treatment [78,79]. Similarly, animal models of neurodegenerative diseases frequently focus on a single pathological mechanism, such as amyloid accumulation, α-synuclein aggregation, or mitochondrial dysfunction, while the pathology of the disorder is much more complex and involves interactions among protein aggregation, neuroinflammation, vascular dysfunction, metabolic disturbances, and age-related changes [45,46,53]. Another important limitation is the time of intervention. In many animal studies, the treatment is initiated before or shortly after disease induction, often during early stages of pathology. In contrast, in humans, especially in neurodegenerative disorders, the diagnosis is made many years after the pathology truly began. It is also worth mentioning that humans (especially the elderly) often have more than one disorder, which leads to questions about possible interactions, both between the drugs and compounds as well as with other disorders. Of note, many animal studies were conducted on relatively small experimental groups and were continued for a relatively short time (a couple of days or weeks), while long-term safety and efficacy data remain unknown. This is a very major restriction, because many disorders, for example epilepsy, involve chronic and long treatment. It should also be taken into account that most of the animal trials compared the INS effect to placebo, and not to currently available and recommended treatments for such a disorder. Although it does not necessarily cross the obtained results out, it leaves a lot of doubt with regard to efficiency and safety. Finally, it should be remembered that modification of disease-related biomarkers does not necessarily translate into clinically meaningful benefits. Despite these limitations, the currently available data should not be interpreted as evidence against the therapeutic potential of SCI, but rather as a need for further, carefully designed clinical studies to fully investigate SCI and other inositols as a potential treatment in neurological disorders.

From a clinical perspective, the development of SCI forms of supplementation should be a point of interest. Currently, in most studies there was a need of supplementation of SCI at least twice a day to achieve steady SCI plasma concentrations [44]. It is currently known that the multi-dose supplementation decreases the patients’ compliance, which can result in missing doses and, in consequence, a non-therapeutic substance plasma concentration, especially in elderly people [124]. Therefore, further studies should concentrate on developing extended-release forms of SCI, which may be supplemented orally once a day or, for example, some depot injection formulations, which may be especially useful in elderly patients with neurodegenerative disorders. An incorporation of SCI into liposomal forms, with increased bioavailability and absorption profile should also be considered in the next clinical trials.

Furter possible SCI studies can also concentrate on different SCI derivates. It is currently known that amino or substituted amino derivatives of INS occur in many antibiotics [27,28]. In recent papers, a new group of insoamino acids (inositol derivatives composed of INS and b- or g-amino acids), is atracting more and more interest due to its usage in the construction of peptide nanotubes with hydrophobic cavities [29]. Peptide nanotubes are currently under intense development due to their unique properties and possible usage in drug and gene delivery, tissue engineering, bio-inspired electronics, or diagnostics [125].

## 8. The “Dark Side” of SCI

During animal and phase I clinal studies, SCI has demonstrated a favorable safety profile, however, in the clinical trials, adverse effects of its supplementation have been observed [22,126]. Although low doses of SCI are well-tolerated [22,44,126,127], the higher doses were shown to cause serious adverse effects. In clinical studies involving Alzheimer’s disease, administration of high SCI doses (up to 2000 mg/day) was associated with an increased incidence of serious adverse events, including respiratory infections, urinary tract complications, and higher mortality rates compared with placebo groups [22]. These observations ultimately contributed to discontinuation of the highest-dose treatment arms and substantially limited further clinical development of SCI at these dosages. In turn, studies on Down syndrome have shown that the administration of SCI leads to disturbances in basic vital parameters [126]; however, it should be noted that high-dose SCI in healthy patients (the control group) does not worsen vital signs [44,126,127].

The mechanisms underlying these adverse effects remain incompletely understood. The interpretation of SCI-associated adverse events is further complicated by the characteristics of the populations enrolled in clinical studies. Most reports of serious adverse effects originated from trials involving patients with chronic neurodegenerative disorders, particularly Alzheimer’s disease and Down syndrome, populations characterized by advanced age, polypharmacy, impaired physiological reserve, and increased baseline susceptibility to infections and renal dysfunction. Consequently, it remains difficult to determine to what extent the observed adverse events were directly attributable to SCI itself rather than to underlying disease processes or interactions between SCI and disease-related physiological alterations. This hypothesis is further supported by the observation that severe adverse events have not been consistently reported in preclinical studies or in clinical populations without significant neurodegenerative burden.

However, the other possible explanations for SCI-associated adverse effects need to be considered. High systemic SCI exposure may exceed physiological renal clearance capacity, leading to altered tubular transport and disturbances in urinary metabolite handling. Other hypothetical mechanisms include osmotic imbalance caused by prolonged accumulation of inositol isomers, altered regulation of urate excretion contributing to hyperuricemia, and disturbances in intracellular signaling pathways involved in immune-cell function. Although these mechanisms remain speculative, they may partly explain why adverse effects have been observed predominantly during long-term administration of high SCI doses in vulnerable patient populations.

Nevertheless, several important questions remain unanswered. The precise dose threshold associated with increased risk has not been established, the biological mechanisms underlying observed adverse effects of SCI supplementation remain poorly characterized, and long-term safety data are limited. Future studies should therefore focus on identifying optimal dosing regimens, characterizing SCI pharmacokinetics in different patient populations, and determining whether specific biomarkers can predict the risk of adverse events.

## 9. Conclusions

SCI has emerged as a promising neuroactive molecule whose unique stereochemistry, pharmacokinetic profile, and multimodal biological effects position it as a candidate for therapeutic modulation of different CNS disorders, especially neurodegenerative disorders and epilepsy. Evidence from preclinical studies demonstrates that SCI penetrates the CNS, engages disease-relevant targets, and exerts neuroprotective actions through mechanisms that include attenuation of amyloid-β aggregation, stabilization of neuronal homeostasis, and modulation of excitatory–inhibitory balance relevant to epileptogenesis. Its pharmacokinetics have been studied both on animals and humans. However, even though a lot is known about SCI pharmacokinetics, long clinical trials have shown that there is still a missing gap, especially in groups of elderly people and SCI doses between 250 and 1000 mg twice a day. Further studies are needed to explore SCI pharmacokinetics and molecular mechanisms of SCI actions. Ultimately, clarifying SCI metabolism will be critical for determining whether SCI can progress from an experimental cyclitol with intriguing potential to a clinically meaningful neuroprotective and antiepileptic agent.

## Figures and Tables

**Table 1:** Summary of SCI in AD.

Authors And Year of Publication	Tested Organism/In Vitro	Route and Dose of SCI Administration	Supplementation Duration	Main Findings
McLaurin et al., 2000 [16]	In vitro	-		Induction of a β-structure transition in Aβ42 that did not lead to fibril formation by SCI
Park et al., 2011 [64]	In vitro	-	--	Inhibition of Aβ aggregation by SCI Decreased oligomer formation by greater than 50% by SCI
Zhao et al., 2011 [65]	In vitro	-	-	Inhibition of Aβ aggregation by SCI
Townsend et al., 2006 [60]	Rat	Orally, 30, 100, and 300 mg/kg/day	>3 days	Reduction in memory impairment produced by soluble Aβ oligomers (administered by Cerebroventricular injection) through a mechanism that restores hippocampal synaptic plasticity
McLaurin et al., 2006 [61]	TgCRND8 mouse model	Orally, 0.3 mg/kg/d to 30 mg/d twice daily	1–4 months	SCI treatment improved AD-like pathology when given prophylactically starting at 6 weeks of age and continuing until 4 and 6 months of age. SCI increased survival from 42% to 72% at 6 months of age and the treated mice showed a complete improvement of cognitive deficits Reduction in synaptic toxicity illustrated by a 146% increase in synaptophysin positive boutons and cell bodies in the hippocampus Reduction in parenchymal plaque load throughout the brain Improved the neuroinflammatory status through the reduction in microgliosis and astrogliosis
DaSilva et al., 2009 [62]	PS1xAPP mouse model	Orally	4 weeks	Reduction in Insoluble Abeta40 and Abeta42 by 20%, in concert with a 50% reduction in plaque load. Restoration of synaptic architecture
Aytan et al., 2013 [63]	5XFAD mouse model	Orally, 3.3 mg/kg/day	1 month	Reduction in the deposition of the amyloid plaques SCI treatment was more effective than the one with SCI + R-flurbiprofen
Salloway et al., 2011 [22]	Human	Orally, 250 mg,1000 mg, 2000 mg twice a daily	78 weeks	Discontinuation of 1000, or 2000 mg SCI doses—imbalance of infections and deaths At the 250 mg dose, CSF Aβx-42 was decreased significantly compared to placebo No significant differences between the treatment groups and placebo on the NTB or ADCS-AD tests

**Table 2:** Summary of inositols action in epilepsy.

Authors And Year of Publication	Tested Organism	Used Inositol and Route of Administration	Main Findings
Wiśniewski et al. (2023) [19]	zebrafish	SCI water solution	short-term exposure to SCI reduced PTZ-treated larva motility in all tested groups
Nozadze et al. (2011) [20]	rat	MI, SCI, orally	significant reduction in seizure score, severity of seizures decreased seizure duration and an increased latency period no difference in mortality rate (placebo vs. treated groups)
Tsverava et al. (2025) [21]	rat	intraperitoneal injections of MI, SCI, DCHI	significant reduction in frequency and duration of SRS (up to 4 weeks from the last dose) SCI and DCHI mitigate the spatial learning and memory deficits associated with epileptogenesis SCI, MI, DCHI supplementation results in protein changes in cells
Kofman et al. (1993) [102]	rat	intracerebroventricular injections of MI	increased latency period and lowered the seizure score
Solomonia et al. (2007) [103]	rat	intraperitoneal injections of MI	decreased seizure activity, increased number of rats exhibiting no epileptic activity
Gamkrelidze et al. (2019) [104]		MI injections into the hippocampus	significant reduction in the discharge duration
Kandashvili et al. (2022) [105]	rat	intraperitoneal injections of MI	decreased the frequency and duration of SRS positive effect on spatial learning and memory deficit decreased cell loss
Tsverava et al. (2019) [106]	rat	intraperitoneal injections of MI	molecular changes in the hippocampus, including mi-RNA expression and enhanced protein expression

## Data Availability

The original contributions presented in this study are included in the article. Further inquiries can be directed to the corresponding author.

## Abbreviations

The following abbreviations are used in this manuscript:

Aβ	amyloid-β
AD	Alzheimer disease
C-C	Carbon–carbon
Cmax	maximum concentration
CNS	central nervous system
DCHI	D-chiro-inositol
DP	D-pinitol
GABA	gamma-aminobutyric acid
INS	inositol
INSs	inositols
KA	kainic acid
MI	myo-inositol
NMDA	N-methyl-D-aspartate
NMR	proton nuclear magnetic resonance
PCOS	polycystic ovary syndrome
PD	Parkinson’s disease
PS1xAPP	APP/PS1 amyloid-beta transgenic mouse model of alzheimer’s disease
PTZ	pentylenetetrazol
SMIT1	Sodium-myo-inositol cotransporter-1
SMIT2	Sodium-myo-inositol cotransporter-2
ZPE	zero-point vibrational energy
5xFAD	5xFAD mice are among the most widely used mouse models of amyloidosis
